# Using tree-based models to identify factors contributing to trait negative affect in adults

**DOI:** 10.1186/s40359-024-02245-z

**Published:** 2025-02-01

**Authors:** Catalina Cañizares, Yvonne Gómez-Maquet, Eugenio Ferro, Carlos Arturo Torres, Diana María Agudelo, Gabriel Odom

**Affiliations:** 1 Florida International University, 11200 SW 8th St, Miami, FL 33199 USA; 2 Universidad de los Andes, Cra. 1 #18a-12, La Candelaria, Bogotá, Cundinamarca Colombia; 3 Instituto Colombiano del Sistema Nervioso Clínica Montserrat, Cl. 134 #17, Usaquén, Bogotá, Colombia

**Keywords:** Negative affect, Adults, Tree-based methods, Depression, Cognitive Schemas

## Abstract

**Background:**

Individuals with high levels of negative affect (NA) are at an increased risk of experiencing distress and negative self-views. Theoretical models suggest that NA plays a critical role in psychopathology, particularly in Major Depressive Disorder (MDD), and is linked to cognitive-perceptual and affective regulation issues.

**Objective:**

Determine whether maladaptive cognitive schemas, attributional style, childhood adversity, and lifestyle factors (including alcohol and drug use and physical activity) could effectively predict negative affect (NA) in adults.

**Methods:**

A secondary data analysis was performed on a sample of 342 depressed and non-depressed adults. Beta regression and regression tree analyses were conducted to identify the principal risk factors and their interactions. The regression tree model was trained with 5-fold cross-validation on 75% of the sample, with 25% of observations held for testing.

**Results:**

The findings revealed that the cognitive schemas of disconnection and rejection and impaired autonomy had a significant impact on the likelihood of higher scores on the State Depression Inventory (IDER) test (*p* < 0.001), as indicated by both beta regression and regression tree analyses. Additionally, childhood adversity emerged as a crucial factor in determining high levels of NA. The regression tree model achieved strong performance metrics, including an R-squared value of 0.77.

**Conclusions:**

This study represents a significant step forward in the understanding of NA, as it considers a broad range of individual factors, such as cognitive schemas, lifestyle, and demographics, to predict its impact on NA, with potential implications for prevention programs aimed at reducing NA.

**Supplementary Information:**

The online version contains supplementary material available at 10.1186/s40359-024-02245-z.

## Background

Major Depressive Disorder (MDD) is a highly prevalent mental disorder worldwide, affecting nearly 280 million people globally and causing significant disability [1]. In Colombia, the most recent data from the National Survey of Mental Health [2] reports a prevalence rate of 4.3% for MDD, 1% for minor depression, and 0.5% for dysthymia, underscoring its prominence in the country. According to classic theoretical frameworks [3] and research [4], Negative Affect (NA) is a central component of MDD. NA refers to the tendency to experience unpleasant emotions, such as anger, guilt, worry, sadness, and disgust [3]. Individuals with high levels of NA are more likely to experience distress and hold a negative self-view, in contrast to those with Positive Affect (PA), who generally exhibit contentment and a positive self-perception [5].

NA has also been linked to specific personality dimensions [6]. Neuroticism is associated with high NA, while extraversion is associated with high PA [6]. Considering NA and PA as part of the dimensional understanding of personality allows for conceptualizing them in terms of both state and trait [6–8]. Trait refers to a relatively stable predisposition to respond in a certain way to situations, while state refers to emotional and mental conditions that are transitory [6]. Therefore, the interaction between state and trait characteristics enables a dimensional understanding of psychopathology, rather than simply considering the frequency of occurrence of symptoms [7, 9]. Therefore, the relationship between NA and psychopathology has been extensively studied, with numerous studies demonstrating a positive relationship between NA and various mental disorders [4, 10].

Despite the established link between NA and psychopathology, research exploring NA’s relationship with other psychological processes, particularly cognition, remains scarce. Specifically, the connection between negative affect and maladaptive schemas has not been extensively examined, and the literature lacks robust experimental or correlational studies to support this relationship. Early theorists posited that maladaptive schemas can lead to NA, manifesting as feelings of sadness, anxiety, and anger, which in turn reinforce these negative beliefs, creating a cycle of negative thinking [11]. Our review of the literature indicates that NA is correlated with various cognitive-perceptual and affective regulation issues [4], including unconventional beliefs, distorted assessments of stimuli, and impaired decision-making [12]. Additionally, high levels of negative affect and early maladaptive schemas have been observed in patients with major depression compared to healthy controls [13]. A pessimistic attributional style has also been found to correlate with negative affect and depressed mood in college students, though this has been less studied in clinical populations [14].

Given the significant impact and relevance of NA in psychopathology, particularly in MDD, several instruments have been developed to measure its presence. Among these, the IDER questionnaire -gets its name from its Spanish title, “Inventario de Depresión Estado Rasgo”- stands out as a valuable tool for evaluating the severity and variability of MDD symptoms, as well as the presence of NA [7, 9]. What sets the IDER questionnaire apart is its ability to measure both state and trait aspects of MDD, with a high level of sensitivity for detecting the presence of NA [7, 9]. It has proven useful in both clinical and research settings, particularly in areas such as personality, emotion, neuropsychology, and cognition [7]. Importantly, the IDER questionnaire focuses on affective symptoms of depression, rather than somatic and physical aspects, making it a measure to evaluate the presence of NA rather than a diagnostic tool [9]. Additionally, the instrument has been tested for its psychometric properties in Colombian samples, demonstrating high levels of reliability (0.71 to 0.86) and confirming the bifactorial structure (dysthymia and euthymia) in exploratory and confirmatory factorial analyses [6].

Considering that theoretical models suggest that NA plays a critical role in psychopathology especially in MDD [3] and MDD correlates with early maladaptative schemas [15] it is essential to identify factors that increase an individual’s likelihood of having higher levels of trait NA. Therefore, in this study, we aim to investigate the extent to which various factors, such as cognitive schemas, attributional style, childhood adversity, and lifestyle factors, including alcohol, drug use, and physical activity, can accurately predict trait NA in a sample of both depressed and non-depressed adults in Colombia using tree-based models.

It is hypothesized that the presence of maladaptive cognitive schemas, negative attributional style, childhood adversity, and lifestyle factors (including alcohol and drug use, and physical activity) will significantly correlate with trait NA. Specifically, maladaptive cognitive schemas and a pessimistic attributional style are expected to be positively associated with higher levels of trait NA. Additionally, greater childhood adversity and higher levels of substance use are anticipated to be positively associated with trait NA, whereas higher levels of physical activity are expected to be negatively associated with trait NA.

Recently, there have been applications of decision tree methods in mental health research given it automatizes detection of main effects and interactions [16]. The advantage of using tree-based models in this study is that they can handle complex relationships between predictors and outcomes, such as intricate interaction effects [17]. Additionally, tree-based models provide an interpretable and intuitive visualization method that can be easily communicated to clinicians [17]. By identifying factors that increase an individual’s likelihood of having higher levels of trait NA, this study may lead to more targeted interventions aimed at reducing NA and improving mental health outcomes in both depressed and non-depressed adults.

## Methods

This study is a secondary data analysis utilizing data collected from a previous study conducted by Lattig and Collegues [18] that investigated the relationship between depression, genetics, and psychosocial variables. By leveraging the existing data, we aim to gain new insights into the factors contributing to NA scores, providing a complementary perspective to the original study.

### Participants

The study recruited a total of 342 individuals, out of which 171 were diagnosed with Major Depressive Disorder (MDD) and recruited from two psychiatric hospitals in Bogota. The diagnosis was confirmed using the DSM IV-TR [19] criteria. To be included in the depressive group, participants had to be inpatients with MDD, over 18 years of age, and have completed at least basic primary education. Those with comorbid substance abuse or dependence, psychotic disorders, or dementia, as well as those with bipolar depression or delirium were excluded. The other 171 were individuals from the general population who had no history of MDD, were over 18 years of age, and had completed basic primary education or higher. Participants were excluded from the control group if they had past or present mental disorders or a family relationship with a case subject. These diagnoses were also confirmed by the Mini-International Neuropsychiatric Interview M.I.N.I [20].

### Measures

The research team created a specialized questionnaire exclusively for this study, aimed at collecting detailed information on demographics, substance use, physical exercise, and exposure to childhood trauma. This questionnaire is original to our research and has not been published elsewhere. The English version can be found in the supplementary materials of this paper.

*Demographics*. The personal questionnaire instrument gathered participants’ sex (male, female) and age.

*Substance use*. The personal questionnaire also collected data on an individual’s alcohol, psychoactive substance, and cigarette usage. Participants were asked to report any usage in the past 30 days and the frequency of their daily consumption.

*Physical Exercise*. The personal questionnaire gathered information on the participants’ exercise routines through questions on their weekly physical activity or sports participation.

*Childhood Adversity*. The personal questionnaire seeked information on the participant’s history of childhood abuse, specifically asking if they have experienced any form of physical and psychologival abuse and the age range in which it occurred.

*Attributional Style*. The Life Experiences Survey (LES) was the tool used to measure an individual’s attributional style in the face of stressful situations. The LES [21] is a 66-item self-report questionnaire designed to evaluate a broad range of stress-inducing life events that an individual has experienced in the past two years. The questionnaire assesses the frequency and perceived stress level of each event, with a rating scale ranging from 0 (not at all stressful) to 4 (highly stressful). Additionally, the LES evaluates the perceived positivity/negativity, expectation, and control of each event. This section uses dichotomous options, where participants classify each stressful event as positive or negative, expected or unexpected, and whether they felt in control or not in control of the situation. The scoring of the test involves summing the responses for the stress scale, resulting in a total stress score and separate totals for each of the dichotomous questions. For the dichotomous questions, a value of 1 is assigned to the response of interest (negative, unexpected, not in control), and a value of 0 is assigned to the positive response (positive, expected, in control). Thus, this scale produces four different scores per participant. The reliability of the LES has been confirmed with a Cronbach’s alpha of 0.82, and it has been shown to have normative data and validity through various studies [21]. In the current sample, Cronbach’s alpha for the total scale was 0.84.

*Cognitive Schemas*. To measure early maladpatative schemas The Young Schema Questionnaire-Short Form (YSQ-SF) was used. The YSQ-SF is 75 items long questionnaire that consists of a subset of 5 items from each of the original 15 scales (the 5 items that load most strongly on the factors derived from factor analysis) [22]. Participants are asked to rate each of the 5 items on a six-point Likert scale, ranging from 1 (completely untrue of me) to 6 (describes me perfectly). Scores for each schema are found by adding the total number of responses within each schema. A study conducted in a Colombian population confirmed the presence of the 15 schemas and demonstrated good psychometric properties, with reported Cronbach’s alpha values ranging from 0.73 to 0.88 for the different schemas [23]. In the current sample, Cronbach’s alpha for the total scale was 0.94.

*State Depression Inventory (IDER)*. The IDER questionnaire is a tool used to assess NA for depression both in terms of its frequency (trait) and intensity (state) at the time of evaluation [7]. It consists of 20 items divided into two scales: Trait and State, each with 10 items, with 5 items for dysthymia and 5 items for euthymia. The subject’s responses are scored based on their chosen answer option, ranging from 1 for “almost never” to 4 for “almost always.” The scores are assigned to items related to dysthymia on both scales. For the items related to euthymia, the score is reversed. The final score of a scale is obtained by summing the results of the two sub-scales, with a range from 20 to 80. The reliability levels of the test are reported to be high, ranging from 0.71 to 0.92 for the different scales in the general population, with Cronbach’s alphas ranging from 0.71 to 0.86 in the Colombian population [6]. In this study, the trait scale was chosen as the outcome measure, high scores range from 40 to 24, average levels range from 22 to 17 and low levels are 16 or less [24]. In the current sample, Cronbach’s alpha for the trait scale scale was 0.94.

### Procedure

The original study was approved by the ethics committees of the participating institutions and an independent research ethics committee. Written informed consent was obtained from those who were interested in participating. The M.I.N.I. structured interview [20] was administered by a trained psychologist on the research team to confirm inclusion criteria and rule out exclusion criteria. Eligible participants then completed a battery of questionnaires, administered by one of the trained psychologists on the research team.

For this study the de-identified dataset was used. This dataset was previously curated to include only the psychosocial and cognitive variables.

### Data analysis

To explore the factors associated with NA, we performed beta regression and regression tree analysis (CART) on the entire dataset without distinguishing between the depressed and non-depressed groups. By analyzing the whole sample as a single group, we were able to capture the full range of possible scores on the IDER scale and examine NA as a construct present in both groups. Considering that NA is a construct that is related to, yet different from, depression given that individuals may experience NA whether or not they are depressed [25].

Both Beta regression and CART were fitted to compare the results using different statistical techniques. Tree-based methods are a type of predictive modeling that seeks to identify similar groups of people based on their outcomes using a series of yes/no questions. These questions are plotted in a graphical format as a tree or bush, with the root representing the initial question and subsequent branches representing subsequent questions. To make predictions for a new person, the tree is traversed from the root to the end of the last branch by answering the yes/no questions. Questions that appear close to the root or frequently throughout the tree are more important for grouping people with similar outcomes.

Tree-based methods can produce very complex trees with small subgroups of people, which can be simplified by pruning or removing certain branches. The optimal amount of pruning is determined by testing different values of the cost complexity parameter (CP) on different parts of the data and choosing the value that results in the best performance on a separate set of data. By simplifying the tree, the model can be made more interpretable and easier to use in practice.

The beta regression model provides a standard approach for mental health audiences to interpret the regression results, while the regression tree output is a useful tool for clinical decision-making as it presents decision heuristics and pathways that clinicians can use when working with clients who have high levels of NA. The beta regression model is suitable for identifying the most significant predictors of NA, while the regression tree model can help clinicians tailor their treatment plans based on individual client needs. Therefore, the two models provide complementary insights that can be used to enhance the understanding and management of NA in mental health settings.

We chose to fit the data in a beta regression considering that the IDER score is conditionally distributed rather than Gaussian [26], meaning it is a continuous variable bounded between 10 and 40. To proceed the analysis we transformed the IDER scores to be between 0 and 1 using the following formula

$$\:{x}_{1}=\frac{{x}_{1}-\text{lower}}{\text{upper}-\text{lower}}$$

where $\:{x}_{1}$ is a specific score, *lower* is the minimum score in the range of scores, in this case 10 and *upper* is the highest score meaning 40.

Given that the data contains values that are at the upper and lower bound it was necessary to apply the lemon squeezer [26] transformation to squeeze the data that lies in [0,1] to be in (0,1). The tranformation applied was

$$\:x{\prime\:}=\frac{x\left(N-1\right)+s}{N}$$

where *N* is the sample size and *s* is a constant between 0 and 1. the *s* acts as if we were taking a prior into account so we decided to choose 0.5 conidering a Bayesian standpoint [26].

The beta regression was fitted using the betareg function from the betareg package in R 4.2.0. The regression tree was fitted using the tidymodels package in R 4.2.0. In preparation for modeling, the data was divided into training (75%) and testing sets (25%) using the initial_split function from the rsample packages. The division of the data was done to ensure that the model was trained on the training set and its performance could be accurately evaluated on the test set. The regression tree model was specified using the r_part engine in regression mode.

**Fig. 1:**
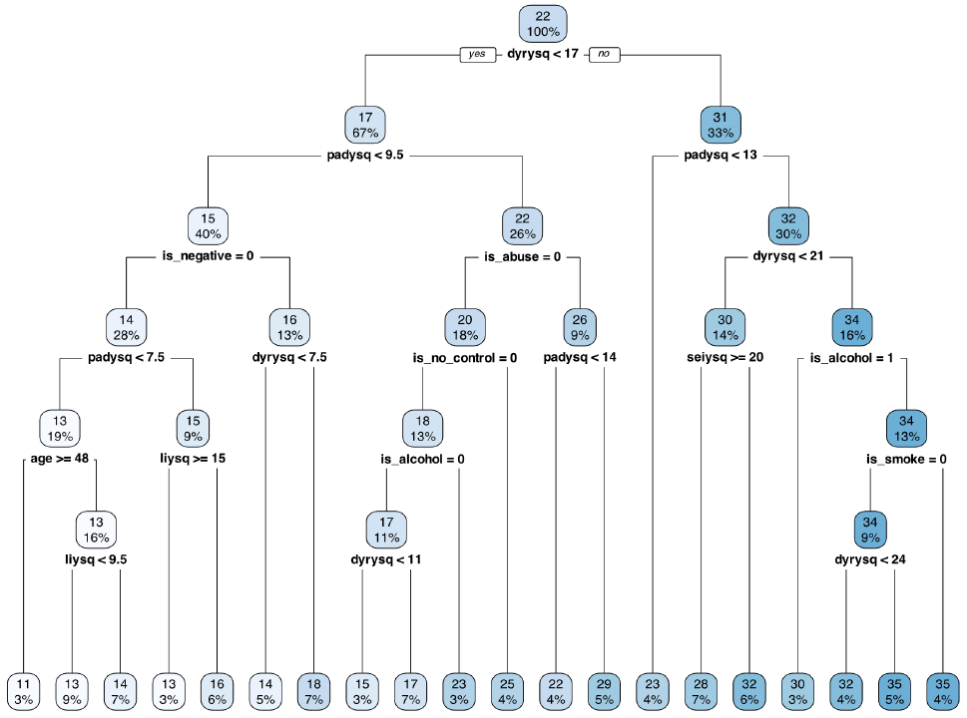
Decision Tree to Predict Negative Affect Score Using the IDER Instrument in a Sample of 255 Depressed and Non-depressed Adults (Training Set)

To avoid overfitting the model, 5-cross-validation was performed using the vfold_cv function from the rsample package. To optimize the model, a grid search was performed to find the best cost complexity parameter (CP). The tune_grid function from the tune package was used to specify a grid of possible cost complexity parameters to test, and the fit function was used to fit the model for each value of the cost CP. The performance metrics, Mean Absolute Error (MAE) and RMSE, were used to compare the results for each value of the CP and select the best model. Finally, the best CP was selected and the final regression tree model was built using the entire training data and was then evaluated on the validation set. All code and analysis scripts are available in the authors GitHub repository. The results of the model fit is displayed in a tree structure (Fig. 1) and summarized using performance metrics.

## Results

### Sample characteristics

Table 1 presents the demographic and lifestyle characteristics of the sample. The sample comprised 249 women and 93 men, with an age range spanning from 18 to 86 years, a mean age of 35.4 years (SD = 11.9), and a median age of 33.0 years. Among the non-depressed participants (*N* = 171), 78.9% were female and 21.1% were male, while among the depressed participants (*N* = 171), 66.7% were female and 33.3% were male. The mean age for non-depressed participants was 36.2 years (SD = 10.8), and for depressed participants, it was 34.6 years (SD = 12.8). In terms of age distribution, the majority of participants were in the early adulthood phase (25–34 years), comprising 37.7% of the sample, followed by young adults (18–24 years) at 19.6%. The mid-adult group (35–44 years) represented 18.7%, mature adults (45–54 years) 15.8%, older adults (55–64 years) 7.3%, and senior adults (65 + years) 0.9%. Childhood adversity was reported by 38.6% of participants, with a significant difference between non-depressed (22.8%) and depressed (54.4%) groups. Regarding lifestyle factors, 39.8% of participants reported engaging in physical exercise, with higher levels among non-depressed participants (45.6%) compared to depressed participants (33.9%). Smoking cigarettes was reported by 19.9% of participants, with a higher incidence in the depressed group (29.8%) compared to the non-depressed group (9.9%). Alcohol use was reported by 14.9% of participants, more common among depressed participants (22.2%) than non-depressed participants (7.6%). Finally, psychoactive substance use was reported by 3.2% of participants, with a higher proportion in the depressed group (5.8%) compared to the non-depressed group (0.6%).

**Table 1:** Demographics and life style variables for 342 depressed and non-depressed participants

	Depressed (*N* = 171)	Non-depressed (*N* = 171)	Overall (*N* = 342)
**Sex**			
Female	114 (66.7%)	135 (78.9%)	249 (72.8%)
Male	57 (33.3%)	36 (21.1%)	93 (27.2%)
**Age**			
Mean (SD)	34.6 (12.8)	36.2 (10.8)	35.4 (11.9)
Median [Min, Max]	32.0 [18.0, 65.0]	33.0 [20.0, 86.0]	33.0 [18.0, 86.0]
**Age Group**			
Young Adults (18–24)	48 (28.1%)	19 (11.1%)	67 (19.6%)
Early Adults (25–34)	54 (31.6%)	75 (43.9%)	129 (37.7%)
Mid Adults (35–44)	24 (14.0%)	40 (23.4%)	64 (18.7%)
Mature Adults (45–54)	29 (17.0%)	25 (14.6%)	54 (15.8%)
Older Adults (55–64)	15 (8.8%)	10 (5.8%)	25 (7.3%)
Senior Adults (65+)	1 (0.6%)	2 (1.2%)	3 (0.9%)
**Childhood Adversity**	93 (54.4%)	39 (22.8%)	132 (38.6%)
**Physical Excercise**	58 (33.9%)	78 (45.6%)	136 (39.8%)
**Smoking Cigarettes**	51 (29.8%)	17 (9.9%)	68 (19.9%)
**Alcohol Use**	38 (22.2%)	13 (7.6%)	51 (14.9%)
**Psychoactive Substance Use**	10 (5.8%)	1 (0.6%)	11 (3.2%)

Table 2 presents the maladaptive cognitive schemas and cognitive attribution scores for the sample of 342 participants, comprising 171 depressed and 171 non-depressed individuals. The IDER score, which indicates the level of maladaptive cognitive schemas, was higher in depressed participants, with a mean score of 28.6 (SD = 6.76) compared to 14.7 (SD = 2.83) in non-depressed participants. The overall mean IDER score was 21.7 (SD = 8.66), with a median score of 19.0. In the domain of Disconnection and Rejection, depressed participants scored a mean of 18.1 (SD = 4.99), while non-depressed participants scored 9.32 (SD = 3.34), with an overall mean of 13.7 (SD = 6.11). For Impaired Autonomy, the mean score was 16.1 (SD = 4.65) for depressed participants and 8.10 (SD = 2.58) for non-depressed participants, resulting in an overall mean of 12.1 (SD = 5.49). In the Impaired Limits domain, depressed participants had a mean score of 18.8 (SD = 4.68), compared to 11.6 (SD = 4.16) in non-depressed participants, with an overall mean of 15.2 (SD = 5.72). The Other-Directedness domain showed a mean score of 19.8 (SD = 4.96) for depressed participants and 12.8 (SD = 3.77) for non-depressed participants, with an overall mean of 16.3 (SD = 5.63). In the Over-Vigilance/Inhibition domain, depressed participants scored a mean of 20.0 (SD = 4.14), while non-depressed participants scored 15.3 (SD = 4.10), with an overall mean of 17.6 (SD = 4.74). Additionally, 84.2% of depressed participants had high negative attribution scores, compared to 31.0% of non-depressed participants. Unexpected attribution was reported by 64.3% of depressed participants and 36.3% of non-depressed participants. Out of control attribution was reported by 38.6% of depressed participants and 4.1% of non-depressed participants. These findings indicate differences in cognitive schemas and attributions between depressed and non-depressed individuals.

**Table 2:** IDER, Maladpatative Cognitive Schemas and Attribution scores for 342 depressed and non-depressed participants

	Depressed (*N* = 171)	Non-depressed (*N* = 171)	Overall (*N* = 342)
**IDER Score**			
Mean (SD)	28.6 (6.76)	14.7 (2.83)	21.7 (8.66)
Median [Min, Max]	30.0 [10.0, 40.0]	15.0 [10.0, 21.0]	19.0 [10.0, 40.0]
**Disconnection and Rejection**			
Mean (SD)	18.1 (4.99)	9.32 (3.34)	13.7 (6.11)
Median [Min, Max]	18.0 [7.00, 29.0]	8.00 [5.00, 21.0]	13.0 [5.00, 29.0]
**Impaired Autonomy**			
Mean (SD)	16.1 (4.65)	8.10 (2.58)	12.1 (5.49)
Median [Min, Max]	16.0 [6.00, 26.0]	8.00 [5.00, 16.0]	11.0 [5.00, 26.0]
**Impaired Limits**			
Mean (SD)	18.8 (4.68)	11.6 (4.16)	15.2 (5.72)
Median [Min, Max]	19.0 [8.00, 30.0]	11.0 [5.00, 23.0]	15.0 [5.00, 30.0]
**Other-Directedness**			
Mean (SD)	19.8 (4.96)	12.8 (3.77)	16.3 (5.63)
Median [Min, Max]	20.0 [8.00, 30.0]	13.0 [5.00, 24.0]	16.0 [5.00, 30.0]
**Over-Vigilance/Inhibition**			
Mean (SD)	20.0 (4.14)	15.3 (4.10)	17.6 (4.74)
Median [Min, Max]	20.0 [11.0, 30.0]	15.0 [6.00, 27.0]	18.0 [6.00, 30.0]
**Negative Attribution**	144 (84.2%)	53 (31.0%)	197 (57.6%)
**Unexpected Attribution**	110 (64.3%)	62 (36.3%)	172 (50.3%)
**Out of Control Attribution**	66 (38.6%)	7 (4.1%)	73 (21.3%)

Higher Scores for the Cognitive Schemas reflect more significant maladaptive schemas. IDER scores: 24–40 High, 17–22 Average, 16 or less Low

### Beta regression

The beta regression model results are presented in Table 3.To verify the model’s assumptions, we visually inspected the beta regression using R’s plot function. The generated graphics revealed that all assumptions were well within the acceptable range, the residuals appeared to be randomly distributed around zero, and the Cook’s distance plot did not indicate the presence of influential observations. Therefore, the beta regression model is valid for interpreting our data. The plots are available at the authors GitHub repository and in the appendix.

We also conducted a variance inflation factor (VIF) analysis to detect multicollinearity in the beta regression model. The results indicated that most predictor variables had VIF values below 3.9, indicating a moderate degree of multicollinearity. However, the Disconnection and Rejection variable had a VIF of 4.9, which could suggest a stronger correlation with other predictor variables in the model. Nevertheless, since none of the VIF values exceeded the recommended threshold of 5 or 10 [27], we concluded that the collinearity was not severe enough to affect our analysis’s overall findings.

**Table 3:** Beta regression for the IDER score in a sample of 255 depressed and non-depressed adults

Predictors	IDER		
	Estimates	CI	*p*
(Intercept)	0.16	0.07–0.35	**< 0.001**
Age	0.99	0.98–1.00	**0.011**
Disconnection and Rejection	1.07	1.03–1.10	**< 0.001**
Impaired Autonomy	1.08	1.05–1.12	**< 0.001**
Impaired Limits	1.02	0.99–1.04	0.126
Other-Directedness	1.00	0.98–1.03	0.939
Over-Vigilance/Inhibition	1.00	0.98–1.03	0.962
Number of Stressful Events	1.00	0.99–1.01	0.785
Sex [Male]	1.07	0.88–1.31	0.477
Negative Attribution [No]	0.66	0.53–0.83	**< 0.001**
Unexpected Attribution [No]	1.04	0.85–1.28	0.675
Out of Control Attribution [No]	0.74	0.59–0.94	**0.012**
Childhood Adversity [No]	0.71	0.59–0.86	**< 0.001**
Physical Exercise [No]	1.03	0.86–1.23	0.773
Smoking Cigarettes [No]	0.75	0.60–0.95	**0.015**
Alcohol Use [No]	0.95	0.74–1.23	0.707
Psychoactive Substance Use [No]	1.43	0.86–2.38	0.165
Observations	342		
R^2^	0.596		

The predictors age, disconnection and rejection, impaired autonomy, negative attribution, out of control attribution, childhood adversity, and smoking cigarettes, are all statistically significant at the 0.05 level, meaning that it is unlikely to have arisen by chance. The negative coefficients for Negative Attribution, Out of Control Attribution, Childhood Adversity, and Smoking Cigarettes suggest that not having these predictors is associated with a decrease in the log odds of experiencing NA (These coefficients are in comparison to the “yes” reference group). In contrast, having these predictors is associated with an increase in the log odds of experiencing NA. Additionally, a negative coefficient for age suggests that as age increases, the odds of experiencing NA decreases.

The results also show that disconnection and rejection and impaired autonomy are significantly associated with the odds of experiencing NA. Each one unit increase in disconnection and rejection increases the log odds of experiencing NA by 0.06 units, while each one unit increase in impaired autonomy increases the log odds of experiencing NA by 0.08 units (in a scale from 0 to 1).

Table 3 displays the precision results for the mean model, which includes the pseudo R-squared of 0.60 that demonstrates a strong fit for the model as noted by Giselmar and collegues [28]. However, it is important to keep in mind that the pseudo R-squared should not be directly compared to the R-squared of linear regression models. This is because the former only measures how well the model fits the data, and does not estimate the accounted variability.

### Regression tree results

Using the selected CP value, the regression tree model achieved an R-squared value of 0.77 on the testing data, indicating that 77% of the variance in the IDER score is explained by the predictors chosen. This substantial R-squared value signifies that the model effectively captures a significant portion of the variance. Moreover, with a Root Mean Square Error (RMSE) of 4.01, the model demonstrates a reasonably accurate prediction capability for the IDER score.

The final decision tree, as shown in Fig. 1, incorporates nine variables, including four cognitive domains: disconnection and rejection (dyrysq), impaired autonomy (padysq), impaired limits (liysq) and over-vigilance/inhibition (seiysq); two attributional styles: negative attribution (is_negative) and out of control attribution (is_no_control); two lifestyle variables: alcohol (is_alcohol) use and smoking cigarettes (is_smoke); and two demographic variables: age and childhood adversity (is_abuse).

The root node of the tree includes 255 observations, with a mean IDER score of 21.88. The disconnection and rejection variable (dyrysq) is the most critical factor in determining high or low IDER scores, with a cutoff value of 17. The tree also features three other main splits based on impaired autonomy (padysq), childhood adversity (is_abuse), and negative attribution (is_negative). These variables are crucial in determining high or low levels of NA.

The decision tree indicates that a score of at least 35 points in the IDER instrument (last blue box to the right) is predicted by the interaction of a 17 or higher score in the disconnection and rejection cognitive domain, a score higher than 21 in the impaired autonomy schema domain, and not consuming alcohol but smoking cigarettes. On the other hand, lower scores are determined by having low scores in the disconnection and rejection schema, lower scores in the impaired autonomy domain, not interpreting stressful situations as negative, and being 48 years of age or older.

In summary, the decision tree depicts several interactions between cognitive, lifestyle, and demographic variables associated with higher levels of NA. Upon analyzing the results of the beta regression and regression tree, it is apparent that there is a correspondence between the two methods, and they provide complementary information. The beta regression demonstrates that cognitive schemas such as disconnection and rejection, and impaired autonomy significantly increase the likelihood of higher scores in the IDER test (*p* < 0.001). These two schemas’ effects are evident in the tree as they are the first two splits, indicating their higher importance in estimating higher levels of NA. The regression tree reveals two additional schemas that are not significant in the beta regression, impaired autonomy and over-vigilance/inhibition. In combination with high levels of disconnection and rejection, impaired autonomy predicts higher scores in the IDER.

Furthermore, both methods agree that childhood adversity’s presence or absence is critical in determining high or low levels of NA. The beta regression suggests that not being subjected to childhood adversity decreases the chances of high scores in the IDER, and the tree regression complements this by showing that low scores are possible in the absence of childhood adversity, but this is only achievable with the presence of interpreting stressful events as under control, not consuming alcohol, and lower scores in the disconnection and rejection schema.

In the end, both methods identify the same predictors as crucial in determining the IDER score’s values, but they complement each other by revealing further interactions. Additionally, the tree delves deeper and shows how alcohol consumption may also be relevant in determining the score.

## Discussion

This study aimed to examine the extent to which cognitive schemas, lifestyle factors, and demographic characteristics could accurately predict NA, as measured by the IDER questionnaire. It used Beta Regression and tree analysis as quantitative methods to evaluate and compare the factors associated with NA in a sample of adults with depression scores ranging from low to high values. Our results indicate that both methods agree on the significant impact of cognitive schemas, attributional styles, lifestyle, and demographic variables in predicting NA. Furthermore, both models demonstrated strong predictive performance, with the regression tree accounting for a considerable proportion of the variance in NA.

These findings suggest that four maladaptive cognitive schemas—namely disconnection and rejection, impaired autonomy, over-vigilance/inhibition and impaired limits—are associated with higher levels of NA. The beta regression analysis showed significant effects only for disconnection and rejection and impaired autonomy, with no significant effect found for impaired limits or over-vigilance/inhibition. Interestingly, the regression tree analysis did capture impaired limits and over-vigilance/inhibition as a maladaptive schema that increases the risk of NA. This discrepancy between the models can be explained by the nature of the regression tree, where the most important splits are at the top. Both disconnection and rejection, as well as impaired autonomy, are among the top splits, indicating their primary importance, which is in concordance with the beta regression. In contrast, impaired limits appears in the fourth split, and over-vigilance/inhibition in the third, suggesting it is not as critical on their own. However, the tree model highlights theit role within interactions, showing that impaired limits and over-vigilance/inhibition becomes important when combined with other factors. This interaction effect provides new insights into the complex dynamics of cognitive schemas in predicting negative affect, that cannot be captured by the linear beta regression model. With generalized regression models, such as the beta regression, there is only a limited amount of information that can be captured by the researcher. This is demonstrated in this results, where the tree, through its interactions, was able to show the importance of other variables in relation to each other in increasing scores of NA.

Introducing new models to analyze social data is essential for the advancement of science. Moving beyond generalized linear models to represent complex real-life phenomena is crucial, and this study exemplifies how supervised machine learning models can be applied to further our understanding of negative affect. These models capture interactions that may be initially unseen by the researcher, thus pushing the boundaries of what is already known.

The results from the beta regression and the tree are consistent with previous research that has shown that the five domains of maladaptive schemas, particularly impaired autonomy and disconnection and rejection, are closely related to the severity of depressive symptoms [29]. This supports the idea that higher NA is related to more persistent and severe symptoms. Therefore, understanding the presence of cognitive schemas is crucial in comprehending the impact of high levels of NA and the corresponding persistence and severity of depressive symptoms.

This study furthers the understanding of cognitive schemas and NA by examining their interactions with other cognitive schemas. While confirming previous findings, it also adds that in the presence of disconnection and rejection, impaired autonomy, over-vigilance/inhibition, and impaired limits, NA scores will increase. Additionally, when these schemas are combined with risk factors such as alcohol and cigarette use, the scores increase significantly more.

The innovation of this study is twofold: It confirms that using both a generalized linear model and a machine learning model yields coherent and similar results, while also providing further information by revealing interactions. This demonstrates the value of incorporating advanced analytical methods to uncover the complex dynamics of cognitive schemas and their impact on negative affect.

Furthermore, this study emphasizes the significance of cognitive schemas over other factors in an individual’s life, as it demonstrates that the most critical factor in determining a higher score was a cognitive schema. While other variables were included in the model after the cognitive schema, this underscores the importance of therapies that aim to improve the flexibility of one’s interpretation and perception of life to achieve better outcomes and reduce negative feelings associated with NA.

The results of this study suggest that negative attribution and the perception of uncontrollability during stressful situations contribute to higher levels of NA. This finding is consistent with previous research that suggests that attributional style is more a product of current mood than a trait-like mode of thinking that increases susceptibility to clinical depression. This is supported by the stronger association between attributional style and current mood found in previous studies [30]. Furthermore, other studies have linked the interpretation of uncontrollability to the severity of depressive symptoms, indicating that the relationship between the perception of uncontrollability and negative affect may be influenced by the level of trait negative affectivity [31, 32].

Another significant finding of this study is the impact of childhood adversity on the likelihood of higher NA. The results showed that childhood adversity was a significant predictor in both models, and the decision tree analysis revealed that being a victim of childhood adversity in association with disconnection and rejection, as well as impaired autonomy, led to a score of approximately 26 in the IDER questionnaire, indicating moderate to high levels of trait NA. This finding aligns with previous research linking childhood adversity to the enduring experience of negative emotions and recurrent and persistent depressive episodes ( [33]). It highlights the importance of early intervention and support for individuals who have experienced childhood adversity to decrease the negative impact on their emotional well-being, overall mental health and ideally, prevention of mental disorders like MDD.

Finally, the results of the study demonstrate a significant relationship between smoking and alcohol use and NA. This finding is consistent with previous evidence showing strong associations between smoking and mental health disorders characterized by persistent NA [34]. Indeed, a large body of literature supports the positive association between smoking status and neuroticism [34], which is a disposition to experience negative affects such as anger and anxiety [35]. As for alcohol use, the tree-based model revealed that it was a relevant factor at two different splits to predict the IDER score. Previous research has shown that higher levels of NA are specifically linked to the initiation and relapse of alcohol and other substance use disorders [36–39].

Moreover, the use of two different quantitative methods to explore the relationship between NA and the introduced predictors is an innovative approach to understanding psychological constructs. This method offers evidence of replicability and internal validity, as both methods agree on most of the significant predictors. Moreover, the tree method provides clinicians with a way to identify which factors related to others are protective or riskier, and represents critical trends that contribute to higher NA scores. This can help clinicians make more informed decisions when designing treatment plans to improve outcomes for patients.

There are several limitations to this study that should be considered. First, it is important to note that this study is a secondary data analysis and the data was not collected specifically to answer the research question being investigated. As a result, the external validity of the study may be limited, as the sample only includes a specific population of depressed individuals and their matched healthy controls. Another limitation is that the data was collected cross-sectionally, which means that causality cannot be established between the predictors and the outcome. When prediction is mentioned, it is in the context of predicting data points into classification, like in a two-by-two table. Another limitation is related to the imbalance between genders; more than half of the sample was composed of women. Although depression has been disproportionately reported by women, in fact, almost twice as often as by men [40], it is relevant to interpret these results with caution for the general population, as the majority are women. Finally, a more comprehensive measurement of psychological and medical factors could help identify other predictors of negative affect and better understand the complex interplay between these factors. Future studies on negative affect should consider longitudinal designs that take into account cultural diversity and include measurements of other psychosocial and medical factors. To improve the generalizability of the findings, future research should aim to use a more diverse sample that includes a wider range of individuals and populations, and a balanced sex ratio. Additionally, longitudinal studies would provide more information on the development and persistence of negative affect over time, which could help identify potential intervention points.

## Conclusions

In conclusion, our study supports and adds to the previous evidence linking NA to cognitive schemas, attributional style, childhood adversity, and lifestyle behaviors by demonstrating the interaction between these predictors. Our model provides a comprehensive picture of how the combination of these variables can accurately predict higher levels of NA thus contributing to a deeper understanding of the development and maintenance of MDD. The findings support the hypothesis that maladaptive cognitive schemas and negative attributional styles are positively associated with higher levels of trait NA.

The results of this study hold significant practical implications for clinical settings. The use of machine learning techniques, as demonstrated in this study, can offer valuable insights for predicting and making decisions by providing clinicians with pathways that lead to more severe levels of NA and possible poor outcomes for the mental disorders it has been related to. If clinicians are provided with these types of tools that contain heuristics specific to particular populations and problems, it could increase awareness of critical risk factors for the persistence and maintenance of depressive disorders. This research represents a significant advancement in the understanding of NA because it takes into account various aspects of an individual, such as their cognitive schemas and interpretations, lifestyle, and demographics, to show that the interaction of these three factors is crucial in determining high levels of NA and, consequently, more persistent symptoms and greater vulnerability to present depression.

## Electronic supplementary material

Below is the link to the electronic supplementary material.

Supplementary Material 1

## Data Availability

The datasets generated and analyzed in the current study are available from the corresponding author on reasonable request.
