# Supplementary material for: Using tree-based models to identify factors contributing to trait negative affect in adults
Source: BMC Psychol. 2025 Feb 1;13:92. doi: 10.1186/s40359-024-02245-z (PMC11786461; doi:10.1186/s40359-024-02245-z)
Supplement: Supplementary file 1 — Supplementary Material 1 [file 40359_2024_2245_MOESM1_ESM.docx]

**DEMOGRAPHIC QUESTIONNAIRE**

**Clinical and family data**

**N0. ________________Date_________________**

SURNAMES_______________________________________________

NAME_________________________________________________

LEVEL OF SCHOOLING __________________________________________________

AGE____________________________________________________

SEX____________________________________________________

MARITAL STATUS____________________________________________

**Family History**

Have brothers/sisters YES _____ NO _____ How many: _____

What place do you have among your siblings ______

Parents' Marital Status

_____ Married _____ Divorced

_____ Separated _____ Common-law

- Lives with:
   **________________________________________________________________**

**_________________________________________________________**

- **Family history of depression**

_____ Dad _______ Mom

_____ Brothers _____ Sisters

_____ Maternal uncles _______ Paternal uncles

______ Maternal grandparents _______ Paternal grandparents

_______ Maternal cousins _______ Paternal cousins

Other_______________

**Family history of psychiatric disorders such as bipolar/schizophrenia**

_____ Dad _______ Mom

_____ Brothers _______ Sisters

_____ Maternal uncles ________ Paternal uncles

______ Maternal grandparents _______ Paternal grandparents

_______ Maternal cousins _______ Paternal cousins

Other_______________

Have you experienced a depressive episode before entering college

YES _____ NO _____

Do you consider yourself a depressed person YES _____ NO_____

**History of stressors**

Were exposed to physical_______ or psychological_______ abuse

_____ Childhood (2-5 years) ________ Childhood (5-12 years)

_____ Adolescence (12-15 years) ____ Adolescence (15-20 years)

Were you exposed to abusive situations?

_____ Childhood (2-5 years) ________ Childhood (5-12 years)

_____ Adolescence (12-15 years) ____ Adolescence (15-20 years)

**HABITS**

You exercise regularly YES _____ NO ______

What sport do you play? _______________________________________________________

Smoke YES ___ NO ___ frequency ______/ week

Drink alcohol: YES ___ NO ___ frequency ____/ week

Use psychoactive substances YES ___ NO ___ frequency ____/ week
